# Fusogenic Liposomes Deliver Resveratrol to Brain Microcirculation and Improve Neurovascular Coupling in Aged Mice

**DOI:** 10.1093/geroni/igaa057.394

**Published:** 2020-12-16

**Authors:** Adam Nyul Toth, Tabea Wiedenhoeft, Stefano Tarantini, Tamas Csipo, Priya Balasubramanian, Anna Csiszar, Agnes Csiszar, Zoltan Ungvari

**Affiliations:** 1 University of Oklahoma Health Sciences Center, Oklahoma City, Oklahoma, United States; 2 Institute of Complex Systems, Jülich, Germany; 3 OUHSC, Oklahoma City, Oklahoma, United States; 4 University of Oklahoma Health Sciences Center, Oklahoma City, United States

## Abstract

Adjustment of cerebral blood flow (CBF) to the increased oxygen and nutrient demands of active brain regions via neurovascular coupling (NVC) has an essential role in maintenance of healthy cognitive function. In advanced age, cerebromicrovascular oxidative stress and endothelial dysfunction impair neurovascular coupling, contributing to age-related cognitive decline. Recently we developed a resveratrol (3,4′,5- trihydroxystilbene)-containing fusogenic liposome (FL-RSV)-based molecular delivery system that can effectively target cultured cerebromicrovascular endothelial cells, attenuating age-related oxidative stress. To assess the cerebromicrovascular protective effects of FL-RSV in vivo, aged (24-monthold) C57BL/6 mice were treated with FL-RSV for four days. To demonstrate effective cellular uptake of FL-RSV, accumulation of the lipophilic tracer dyes in cells of the neurovascular unit was confirmed using two-photon imaging (through a chronic cranial window). NVC was assessed by measuring CBF responses (laser speckle contrast imaging) evoked by contralateral whisker stimulation. We found that NVC responses were significantly impaired in aged mice. Treatment with FL-RSV significantly improved NVC responses by increasing NO-mediated vasodilation. These findings are paralleled by the protective effects of FL-RSV on endothelium-dependent relaxation in the aorta. Thus, treatment with FL-RSV rescues endothelial function and NVC responses in aged mice. We propose that resveratrol containing fusogenic liposomes could also be used for combined delivery of various anti-geronic factors, including proteins, small molecules, DNA vectors and mRNAs targeting key pathways involved in microvascular aging and neurovascular dysfunction for the prevention/treatment of age-related cerebromicrovascular pathologies and development of vascular cognitive impairment (VCI) in aging.

